# Enhanced expression of codon optimized *Mycobacterium avium* subsp. *paratuberculosis* antigens in *Lactobacillus salivarius*

**DOI:** 10.3389/fcimb.2014.00120

**Published:** 2014-09-04

**Authors:** Christopher D. Johnston, John P. Bannantine, Rodney Govender, Lorraine Endersen, Daniel Pletzer, Helge Weingart, Aidan Coffey, Jim O'Mahony, Roy D. Sleator

**Affiliations:** ^1^Biological Sciences Department, Cork Institute of TechnologyCork, Ireland; ^2^United States Department of Agriculture - Agricultural Research Service, National Animal Disease CenterAmes, IA, USA; ^3^School of Engineering and Science, Jacobs University BremenBremen, Germany

**Keywords:** MAP antigens, MptD, MMP, codon optimization, expression host, *paratuberculosis*, MAP vaccine, Johne's disease

## Abstract

It is well documented that open reading frames containing high GC content show poor expression in A+T rich hosts. Specifically, G+C-rich codon usage is a limiting factor in heterologous expression of *Mycobacterium avium* subsp. *paratuberculosis* (MAP) proteins using *Lactobacillus salivarius*. However, re-engineering opening reading frames through synonymous substitutions can offset codon bias and greatly enhance MAP protein production in this host. In this report, we demonstrate that codon-usage manipulation of MAP2121c can enhance the heterologous expression of the major membrane protein (MMP), analogous to the form in which it is produced natively by MAP bacilli. When heterologously over-expressed, antigenic determinants were preserved in synthetic MMP proteins as shown by monoclonal antibody mediated ELISA. Moreover, MMP is a membrane protein in MAP, which is also targeted to the cellular surface of recombinant *L. salivarius* at levels comparable to MAP. Additionally, we previously engineered MAP3733c (encoding MptD) and show herein that MptD displays the tendency to associate with the cytoplasmic membrane boundary under confocal microscopy and the intracellularly accumulated protein selectively adheres to the MptD-specific bacteriophage fMptD. This work demonstrates there is potential for *L. salivarius* as a viable antigen delivery vehicle for MAP, which may provide an effective mucosal vaccine against Johne's disease.

## Introduction

*Mycobacterium avium* subsp. *paratuberculosis* (MAP) is an intracellular pathogen and the etiological agent of Johne's disease, a chronic inflammatory disorder of the gastrointestinal tract which affects multiple ruminant species including cattle (Chacon et al., 2004). Live attenuated vaccine formulations against Johne's disease do not appear to offer adequate protection against MAP infection in goats (Hines et al., 2014) and while heat-killed whole cell vaccines that are commercially available do show some efficacy, these also fail to provide full protection against MAP in models of infection (Rosseels and Huygen, 2008). Moreover, issues relating to interference with diagnostic assays for bovine tuberculosis have further hindered their widespread development and application (Buddle et al., 2010; Bastida and Juste, 2011; Coad et al., 2013). These limitations combined with the availability of complete genome sequences for MAP (Li et al., 2005; Bannantine et al., 2014) have focused recent attention on experimental subunit based vaccine strategies against MAP (Bull et al., 2007; Rosseels and Huygen, 2008). One particularly promising subunit vaccine for MAP is the 70 kDa heat shock protein termed Hsp70 (Koets et al., 2006), which activates B cells (Vrieling et al., 2013).

*Lactobacillus* are interesting candidates for the development of novel oral vaccine vectors due to certain strains widespread use in the food industry and GRAS (generally regarded as safe) status (Yu et al., 2013). Specific members of this genus are also an attractive alternative to using attenuated pathogens for mucosal delivery strategies because they can survive the upper gastrointestinal tract (GI) and colonize the lower GI tract (Bermudez-Humaran et al., 2011). Numerous studies have substantiated the potential of specific *Lactobacilli* strains to serve as live vaccine delivery vehicles against a broad spectrum of mucosal pathogens including *Bacillus anthracis*, *Streptococcus pneumonia, Clostridium difficile* and the Avian Influenza Virus H5N1 (Campos et al., 2008; Sleator and Hill, 2008; Mohamadzadeh et al., 2010; Wang et al., 2012). MAP is particularly well-suited to *Lactobacillus* vector delivery because it is a pathogen transmitted by ingestion of contaminated feces or milk and passes through the GI tract where it infects the intestinal mucosa (Bannantine and Bermudez, 2013).

However, despite the obvious advantages of *Lactobacillus* based mucosal immunization; encompassing the inherent ability of specific strains to survive gastric transit, adhere to the intestinal epithelium (Messaoudi et al., 2012), and stimulate both mucosal and systemic immune responses (Mohamadzadeh et al., 2009), there are known difficulties in expression of G+C rich coding sequences in the A+T rich *Lactobacillus* host (Johnston et al., 2013). Indeed, we recently determined that the significantly divergent genomic G+C content of MAP and *Lactobacillus salivarius* (69 and 33% respectively) leads to a disparity in codon usage, which significantly impedes recombinant MAP protein synthesis. To alleviate this translation inefficiency likely due to ribosomal pausing at rare codons (Buchan and Stansfield, 2007), codon optimization of a MAP gene (MAP3733c) was performed by introducing a series of synonymous mutations; modifying the coding region to better suit the codon bias of *L. salivarius*. It was shown that synthesis of a MAP specific membrane protein within *L. salivarius* could be markedly enhanced (>37-fold) owing to codon optimization, resulting in the abundance of MAP-GFP protein fusion fluorescence in recombinant cells (Johnston et al., 2013). However, that protein was never confirmed to truly represent the native protein as no monoclonal antibodies or other specific detection reagents were developed against it.

Because MAP surface proteins likely play the dominant role in the initial interactions with bovine intestinal cells (Bannantine et al., 2003; He and De Buck, 2010; Gurung et al., 2012), we here focused on two important MAP membrane proteins, MMP (MAP2121c) and the previously studied MptD (MAP3733c). MMP, encoded by MAP2121c, is a ~33.5 kDa surface protein which shares homology to a *Mycobacterium leprae* 35-kDa major membrane protein-1 (MMP-1) (Winter et al., 1995), previously identified as a potent immunodominant antigen capable of inducing T-lymphocyte responses in paucibacillary leprosy patients (Triccas et al., 1996). MptD is a MAP specific, virulence associated membrane protein which is surface expressed during natural infection, warranting its further investigation as a prophylactic antigen (Stratmann et al., 2004; Shin et al., 2006; Cossu et al., 2011).

A significant limitation of current whole cell MAP vaccine strategies is interference with cellular immune assays and tuberculin skin tests for bovine tuberculosis (*M. bovis*), which restricts their widespread application in many countries (Kohler et al., 2001; Scandurra et al., 2010). It is notable that although MMP is not specific to MAP, with homologs existing in other mycobacterial species including *M. avium* (MAC) and *M. leprae*, previous genetic and serological evidence suggests that the protein is absent from *M. tuberculosis* and *M. bovis* (Triccas et al., 1998; Banasure et al., 2001). In addition, a DNA vaccine incorporating the *M. leprae* MMP-1 protein in isolation demonstrated significant levels of protective efficacy against *M. leprae* footpad infection in outbred Swiss Albino mice (Martin et al., 2001). As such, if an MMP-based vaccine against Johne's disease were to display strong prophylactic efficacy, as it has done against Leprosy, it leads to the exciting prospect of a vaccine formulation that could overcome issues with interference of bovine tuberculosis screening assays.

Herein, we analyzed MAP proteins recoded using synonymous substitutions and expressed in *L. salivarius*. Faithful expression and antigenicity was examined using a combination of fluorescence microscopy, monoclonal antibody- and bacteriophage-based ELISA.

## Materials and methods

### Strains, plasmids, bacteriophage, and growth conditions

The strains and plasmids used in this study are listed in Table 1. *Mycobacterium avium* subsp. *paratuberculosis* strain K-10 (ATCC BAA968) were propagated in Middlebrook 7H9 broth supplemented with OADC (BD Biosciences), 2 μg/ml of Mycobactin J and 0.2% glycerol and incubated at 37°C for 6–10 weeks. *Escherichia coli* DH5α electrocompetent cells (Invitrogen) were used as intermediate cloning hosts for all vector constructs within this study and grown at 37°C in Luria-Bertani (LB) media. *Lactobacillus salivarius* NRRL B-30514 (kindly donated by Dr. Norman Stern, USDA), was used throughout this study as the host for individual vector constructs and was grown aerobically at 37°C in MRS media (Fluka).

**Table 1:** **Strains, Phage, and Plasmids used in this study**.

**Plasmid, phage, and strains**	**Relevant genotype or characteristics**	**Source or References**
**PLASMIDS**		
pNZ9530	N.I.C.E system helper plasmid. Ery^r^, *nisRK* cloned in pIL252, expression of *nisRK* driven by *rep* read through, low copy number	Pavan et al., 2000
pNZ8048	N.I.C.E system expression plasmid. Cm^r^, carries the nisin-inducible promoter P*nisA*	Pavan et al., 2000
pNZ:MAP3733c	pNZ8048 with MptD fused to GFP gene under control of P*nisA* promoter	This work
pNZ:MAP3733synth	pNZ8048 with codon optimized MptDsynth gene under control of P*nisA* promoter	This work
pNZ:MAP3733c-GFP	pNZ8048 with MptD fused to GFP gene under control of P*nisA* promoter	Johnston et al., 2013
pNZ:MAP3733synth-GFP	pNZ8048 with codon optimized MptDsynth fused to GFP gene under control of P*nisA* promoter	Johnston et al., 2013
pNZ:MAP2121c	pNZ8048 with MMP fused to GFP gene under control of P*nisA* promoter	This work
pNZ:MAP2121synth	pNZ8048 with codon optimized MMPsynth gene under control of P*nisA* promoter	This work
pNZ:MAP2121c-GFP	pNZ8048 with MMP fused to GFP gene under control of P*nisA* promoter	This work
pNZ:MAP2121synth-GFP	pNZ8048 with codon optimized MMPsynth fused to GFP gene under control of P*nisA* promoter	This work
pNZ:GFP	pNZ8048 with *L. salivarius* codon optimized GFP gene under control of P*nisA* promoter	Johnston et al., 2013
**PHAGE**		
Phage fMptD	Phage isolated from the Ph.D.-12 phage display library, with a dodecapeptide sequence (GKNHHHQHHRPQ) fused to the N-terminus of its minor coat protein (pIII)	Stratmann et al., 2004
**STRAINS**		
***Mycobacterium avium* subsp. *paratuberculosis***		
MAP K-10	American bovine virulent isolate and sequencing project reference strain	Li et al., 2005
***Escherichia coli***		
DH5α	Intermediate cloning host	Invitrogen
***Lactobacillus salivarius***		
NRRL B-30514	Host strain, originally isolated from cecal contents of broiler chicken. Aerobic	Stern et al., 2006
*L. salivarius* pNZ9530	Host strain harboring pNZ9530 helper plasmid, Ery^r^	Johnston et al., 2013
*L. salivarius* pNZ8048	Harbors pNZ9530 plasmid and pNZ8048 expression plasmid lacking insert. Ery^r^ and Cm^r^	Johnston et al., 2013
*L. salivarius* MAP3733c	Harbors pNZ9530 and pNZ:MAP3733c plasmids. Ery^r^ and Cm^r^	This work
*L. salivarius* MAP3733synth	Harbors pNZ9530 and pNZ:MAP3733synth plasmids. Ery^r^ and Cm^r^	This work
*L. salivarius* MAP3733c-GFP	Harbors pNZ9530 and pNZ:MAP3733c-GFP plasmids. Ery^r^ and Cm^r^	Johnston et al., 2013
*L. salivarius* MAP3733synth-GFP	Harbors pNZ9530 and pNZ:MAP3733synth-GFP plasmids. Ery^r^ and Cm^r^	Johnston et al., 2013
*L. salivarius* MAP2121c	Harbors pNZ9530 and pNZ:MAP2121c plasmids. Ery^r^ and Cm^r^	This work
*L. salivarius* MAP2121synth	Harbors pNZ9530 and pNZ:MAP2121synth plasmids. Ery^r^ and Cm^r^	This work
*L. salivarius* MAP2121c-GFP	Harbors pNZ9530 and pNZ:MAP2121c-GFP plasmids. Ery^r^ and Cm^r^	This work
*L. salivarius* MAP2121synth-GFP	Harbors pNZ9530 and pNZ:MAP2121synth-GFP plasmids. Ery^r^ and Cm^r^	This work
*L. salivarius* GFP	Harbors pNZ9530 and pNZ:GFP plasmids. Ery^r^ and Cm^r^	Johnston et al., 2013

Plasmids pNZ9530 and pNZ8048 were originally obtained from the University College Cork culture collection, while pUC57 vectors containing codon-optimized synthetic GFP and MptDsynth genes were obtained from the Cork Institute of Technology culture collection (Johnston et al., 2013). Cultures of *E. coli* harboring individual vectors were supplemented with Erythromycin (Ery, 200 μg/ml), Chloramphenicol (Cm, 12.5 μg/ml), or Ampicillin (Amp, 200 μg/ml) for pNZ9530, pNZ8048, and pUC57 containing cells respectively. Recombinant *L. salivarius* cells were subcultured from 40% v/v glycerol stocks at −20°C and grown using antibiotic selection. *L. salivarius* (pNZ9530) cultures were supplemented with 5 μg/ml Ery, while *L. salivarius* harboring both pNZ9530 and pNZ8048 constructs were grown in the presence of 5 μg/ml Ery and 10 μg/ml Cm.

The M13 phage fMptD, originally isolated from the Ph.D.−12 phage display library (New England Biolabs), was obtained from the laboratory of Gerald F. Gerlach (University of Veterinary Medicine, Hanover, Germany). Bacteriophage fMptD was propagated using standard methods previously described (Chappel et al., 1998). Phage titers were assessed by a standard plaque assay test (Sambrook et al., 1990). Purified high titer phage solutions (10^10^ pfu/ml) were stored at 4°C until required.

### Nucleic acid isolation

Mycobacterial DNA was prepared as described previously by Douarre et al. (2010). Plasmid DNA was isolated from *E. coli* DH5α using a plasmid extraction kit as per the manufacturer's instructions (Sigma Aldrich). Plasmid isolation from *Lactobacillus* strains was also carried out using this kit, after initial incubation (30 min at 37°C) in protoplast buffer (20 mM Tris-HCl, 5 mM EDTA, 0.75 M Sucrose, 10 mg/ml lyzozyme and 50 U/ml mutanolysin pH 7.5). Quantitative analysis was carried out using microspectrophotometry (Nanodrop, De, USA) and plasmid DNA concentration was normalized to 250 ng/μl.

### Codon optimization of MAP2121c

MAP2121c encodes a major membrane protein (MMP) in MAP. Because this protein has been implicated in the early events of infection in the bovine intestinal musoca, it is an ideal candidate for testing expression in *L. salivarius* as a potential mucosal vaccine. We have previously described a strategy for the codon optimization of the MAP3733c gene for expression in *L. salivarius* (Johnston et al., 2013). Here we used the same strategy for MAP2121c; briefly, a bioinformatics analysis was performed to identify codons from MAP2121c that could be modified at the third base position without a change in the resulting amino acid (termed a synonymous substitution). Coding sequences were synthesized by GenScript USA Inc. (Piscataway, NJ). Constructs were cloned as described below and confirmed by DNA sequencing. Final sequences for each gene are available from GenBank (Accession numbers: KC854397 and KC517484). All modifications to MAP2121c are summarized in Table 2.

**Table 2:** **Modification and codon optimization of MAP genes**.

**Gene**	**Length (bp)**	**G+C content**	Unfavorable codons^*^	**Modified codons**	**Source**
MAP2121c	924	66.5%	276/307	0/307	This work
MAP2121synth	924	32.8%	7/307	279/307	This work

* Codons were deemed unfavorable due to the presence of a guanine or cytosine within the 3rd base of the triplet.

### PCR amplifications and modifications

PCR primers are listed in Supplementary Table S1. Primers were designed for the native and codon optimized MAP genes (MAP2121c, MAP3733c, MAP2121synth, and MAP3733synth) based on either MAP strain K-10 sequence data available from the NCBI database (NC_002944) or using the sequence from GenScript synthesized genes. All conventional PCR reactions were carried out using high fidelity Velocity DNA polymerase Kit (Bioline) in accordance with the manufacturer's instructions. Restriction enzymes and T4 DNA ligase were purchased from Roche Diagnostics (Mannheim, Germany) and New England Biolabs (Beverly, MA, USA) and used as per manufacturer's recommendations. Ligation reaction mixtures were purified using the High Pure PCR product purification kit (Roche).

### MAP gene and fusion constructs

Individual MAP gene constructs (native and synthetic) were cloned into the *E. coli*-*L. lactis* shuttle vector pNZ8048, a derivative of pNZ124 that allows expression of proteins under the control of the nisin-inducible promoter *PnisA*, part of the NIsin Controlled Expression (NICE) system (Pavan et al., 2000). Nisin induced expression was achieved in *L. salivarius* via co-transformation of a dual plasmid system; pNZ8048 with insert to be expressed downstream of *PnisA* and pNZ9530 providing the necessary *nisRK* regulatory genes in *trans* (de Ruyter et al., 1996).

Two native MAP genes, MAP2121c and MAP3733c (designated “c” to indicate that each is originally transcribed on the complimentary strand of the MAP K-10 genome) and two synthetic codon optimized counterparts of these, designated MAP2121synth and MAP3733synth, were initially cloned forming pNZ:2121c, pNZ:3733c, pNZ:2121synth, and pNZ:3733synth. To facilitate fluorometric analysis of each of these genes during expression and monitor subcellular localization of proteins within the host cell, a C-terminus GFP gene was translationally fused to each gene construct forming pNZ:2121c-GFP, pNZ:3733c-GFP, pNZ:2121synth-GFP, and pNZ:3733synth-GFP. The fusion of GFP and individual MAP genes was performed using Splicing by Overlap Extension (SOEing) as previously described (Johnston et al., 2013).

The GFP coding region used for fusions throughout this study was codon optimized for use within *L. salivarius*. The GFP gene sequence cloned into pNZ8048 (Johnston et al., 2013) was used to provide a comparative control for subsequent assays and designated pNZ:GFP.

### Transformation and induction of *L. salivarius* constructs

Competent *L. salivarius* (pNZ9530) were transformed individually with each of the MAP gene constructs (Table 1) as described previously (Johnston et al., 2013). Overnight cultures of recombinant *L. salivarius* strains were subcultured (1:100 dilution) into fresh MRS broth (Cm 8 μg/ml, Ery 3.5 μg/ml) and incubated with agitation (100 rpm) at 37°C. At an optical density (OD_600nm_) of 0.35, nisin was added at a final concentration of 10 ng/ml and cultures were incubated statically at 37°C for 2 h. 10 ml aliquots of each culture were then harvested by centrifugation (6000 rpm for 5 min) for subsequent analysis.

### Fluorescence microscopy

To facilitate visualization of cells using fluorescence microscopy, induced *L. salivarius* strains (*L.sal* GFP, *L.sal* MAP2121c-GFP, *L.sal* MAP2121synth-GFP, *L.sal* MAP3733c-GFP, *L.sal* MAP3733synth-GFP) were fixed using 3.7% formaldehyde, washed with PBS (pH 7.2), subsequently resuspended in 1 ml PBS and stored at 4°C until visualized. For detection of GFP fusion peptides, *L. salivarius* cell images were taken using a Zeiss LSM 510 META laser-scanning microscope equipped with Argon and Helium-Neon lasers (Carl Zeiss, Oberkochen, Germany) at a resolution of 2048 × 2048 pixels, using LSM 5 software (version 3.2; Carl Zeiss). Equal settings were used for detection of green fluorescence among different strains (Amplifier Offset: 0.05, Amplifier Gain: 1, Gain: 820).

### Preparation of whole cell *L. salivarius* for ELISA

To determine if recombinant MMP and MMPsynth peptides were displayed on the surface of whole cell *L. salivarius*, nisin-induced strains were harvested, washed and resuspended in PBS and coated directly to the wells of a Nunc Maxisorp plate (1 × 10^9^ cells/ml). To determine if these peptides were accumulating within the cytoplasm of *L. salivarius*, 500 μl of the harvested cells were transferred to 2 ml screw cap tubes with 0.3 g glass beads (Sigma, 150–212 μm, acid washed) and lysed (4000 rpm for 45 s) using a MagNA Lyser Instrument (Roche). Subsequently, 100 μl aliquots of crude cell lysate were added to individual wells of the same Maxisorp plate.

In the analysis of MptD proteins, similar techniques were applied for whole-cell and cell-lysate preparations, however, use of the alternative bacteriophage (fMptD) detection method necessitated the substitution of PBS for TBS in all washing and subsequent steps. MAP strain K-10, processed in the same manner, was included in all assays to provide a comparative control.

### Monoclonal antibody based ELISA of MMP

Maxisorp plates containing recombinant *L. salivarius* (pNZ, MMP, MMPsynth, MMP-GFP, and MMPsynth-GFP), as well as MAP K-10, whole cells and cell lysate were incubated at 37°C for 1 h and then blocked with a 5% (w/v) solution of dry skimmed milk powder in PBS. A 100-μl aliquot of purified monoclonal antibody 13E1 or 8G2, appropriately diluted in PBS plus 0.1% tween 20 (PBS/T) was added to each test well. Samples were incubated at 37°C for 1 h on a rocking platform. The wells were washed and 100 μl of secondary antibody (Peroxidase-labeled, Anti-Mouse IgG detection antibody) diluted in PBS/T containing 1% (w/v) milk powder was added. After a 1 h incubation at 37°C the wells were washed and 100 μl of 3,3′,5,5′-Tetramethylbenzidine Liquid Substrate System for ELISA (Sigma) was added per well. The substrate was left to develop in the dark at room temperature for 30 min after which the reaction was stopped by addition of 50 μl of a 10% HCl solution. Absorbance readings were read at 450 nm using a microplate reader. The binding response values against each respective recombinant cell type were normalized by dividing the absorbance level obtained in test wells by that obtained in parallel control wells treated with diluent buffer without the addition of the primary monoclonal antibody.

### Bacteriophage-fMptD based ELISA of MptD

Due to a lack of monoclonal antibodies available for the MptD protein, the fMptD bacteriophage (Stratmann et al., 2006) was used in lieu of the primary binding antibody within MptD ELISA. Maxisorp plates containing recombinant *L. salivarius* (pNZ, MptD, MptDsynth, MptD –GFP, and MptDsynth-GFP), as well as MAP K-10, whole cells and cell lysate were incubated at 37°C for 1 h and then blocked with a 5% (w/v) solution of dry skimmed milk powder in TBS. Plates were washed with TBS/T (TBS + 0.1% [v/v] Tween-20) and 100 μl of fMptD (10^9^ pfu/ml) diluted in TBS/T was added to each test well after which samples were incubated at 37°C for 1 h on a rocking platform. Individual wells were subsequently washed and 100 μl of the detection antibody, HRP Conjugated anti-M13 monoclonal antibody (GE Healthcare), diluted in TBS/T containing 1% (w/v) milk powder was added. After 1 h incubation at 37°C the wells were washed, 100 μl of TMB Liquid Substrate (Sigma) was added per well and color developed as already described.

### Statistical analysis

Statistical analysis was carried out using GraphPad Prism (version 4.03; GraphPad Software, San Diego, CA). Means with standard error (s.e.m.) are presented in each graph. Differences between two groups were calculated using unpaired Student's *t*-test. Differences were considered significant at *P* < 0.05.

## Results

### Distinct localization of MAP-GFP fusion peptides

**Figure 1:**
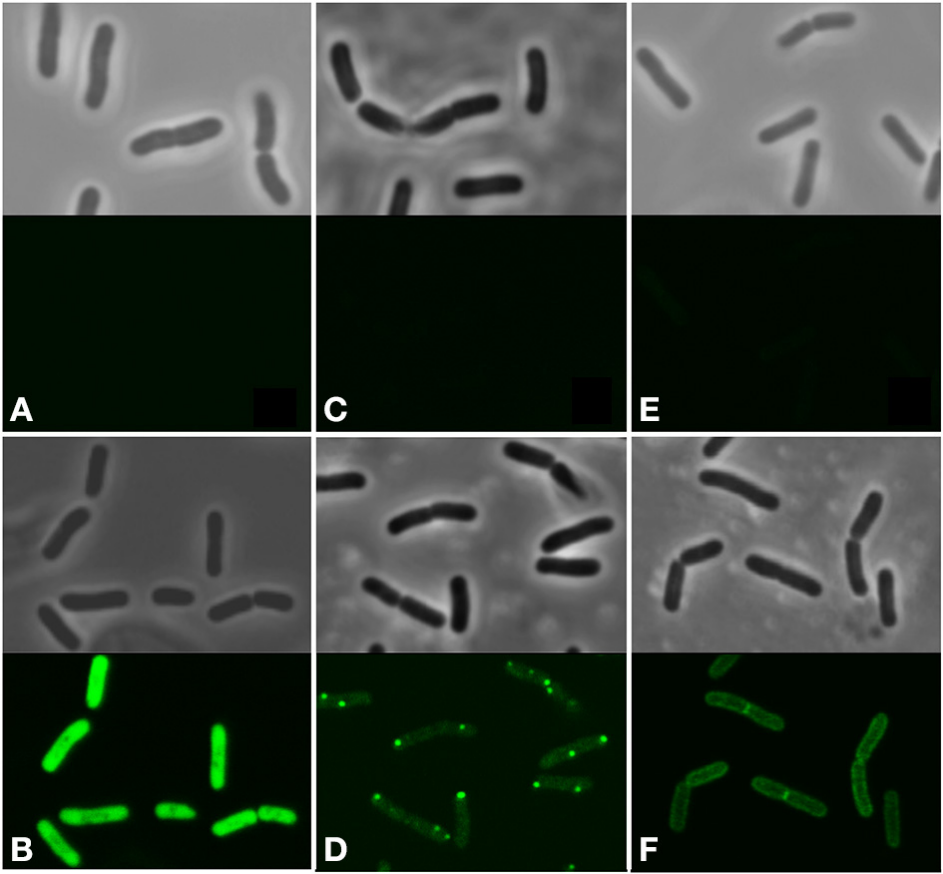
**Representative phase contrast (upper panel) and fluorescent microscopy (lower panel) images of recombinant *L. salivarius* cells. (A)** Control cells pNZ:8048. **(B)** *L. salivarius* GFP cells displayed fluorescence observed throughout individual bacilli. **(C)** Negligible levels of fusion fluorescence were observed from MMP-GFP cells, **(D)** while fluorescent foci, suggestive of aggregation, were observed toward the polar regions of individual cells for the codon optimized variant MMPsynth-GFP. **(E)** Low levels of fluorescence prevented determination of the subcellular localization of native MptD-GFP fusions within *L. salivarius* cells, while **(F)** codon optimized MptDsynth-GFP fusion proteins demonstrated the tendency to localize toward the cellular membrane periphery. All culture assays were performed in triplicate, multiple images were taken from each sample and representative pictures were chosen. Bars represent 5 μm.

In our previous work we confirmed poor fluorescence for the native MptD-GFP fusion protein expressed in *L. salivarius*, and showed markedly improved expression through codon optimization of the synthetic gene (MptDsynth-GFP; Figures 1E,F) (Johnston et al., 2013). Here, consistent with our previous findings for MptD, induction and confocal microscopy of the native MMP-GFP fusion resulted in no fluorescence (Figure 1C) similar to the *L. salivarius* wild type control (Figure 1A). In contrast, improved levels of fluorescence were noted for codon optimized MMPsynth-GFP after induction under identical conditions as the native variant (Figure 1D).

While an even distribution of strong fluorescence was observed within pNZ:GFP-containing bacilli (Figure 1B), both engineered MAP fusion displayed different fluorescence patterns when expressed within *L. salivarius* (Figures 1D,F). MMPsynth-GFP was aggregated (Figure 1D) whereas MptD was more uniformly distributed around the periphery of the cells (Figure 1F).

#### MptD-GFP (MAP3733c/synth)

*In silico* TMHMM analysis of MptD suggests the presence of six transmembrane segments (TMSs) within the 208 amino acids and a large external loop at positions 147–175, with both N and C termini being cytoplasmic associated. In cases where transmembrane proteins have their native C-terminus located in the cytoplasm, fusion of a GFP tag is particularly useful for analysis of protein localization. If the fusion is expressed at the cytoplasmic membrane the GFP peptide may fold correctly and fluoresce allowing visualization of protein localized at this membrane, however if it aggregates and forms inclusion bodies the downstream GFP might not fold correctly and therefore not fluoresce (Drew et al., 2005), although this will be largely protein dependant.

The MptDsynth-GFP fusion peptides demonstrated a tendency to localize at the periphery of individual cells (Figure 1F), suggestive of membrane domain insertion and conformation. This result extends our initial findings with MptD (Johnston et al., 2013).

#### MMP-GFP (MAP2121c/synth)

Confocal microscopy of *L. salivarius* MMP-GFP resulted in undetectable fluorescence from individual bacilli (Figure 1C), further supporting our previous hypothesis that native MAP genes are poorly translated within *L. salivarius*. However, fluorescent foci were observed within the re-engineered sequence (MMPsynth-GFP) suggesting the presence of aggregated GFP fusion proteins within the cytoplasm of *L. salivarius* (Figure 1D). This result was not expected since MMP was predicted to be more soluble than MptD using SOLpro1.0 software (Magnan et al., 2009).

### Recombinant MptD accumulates within the cytoplasm of *L. salivarius*

**Figure 2:**
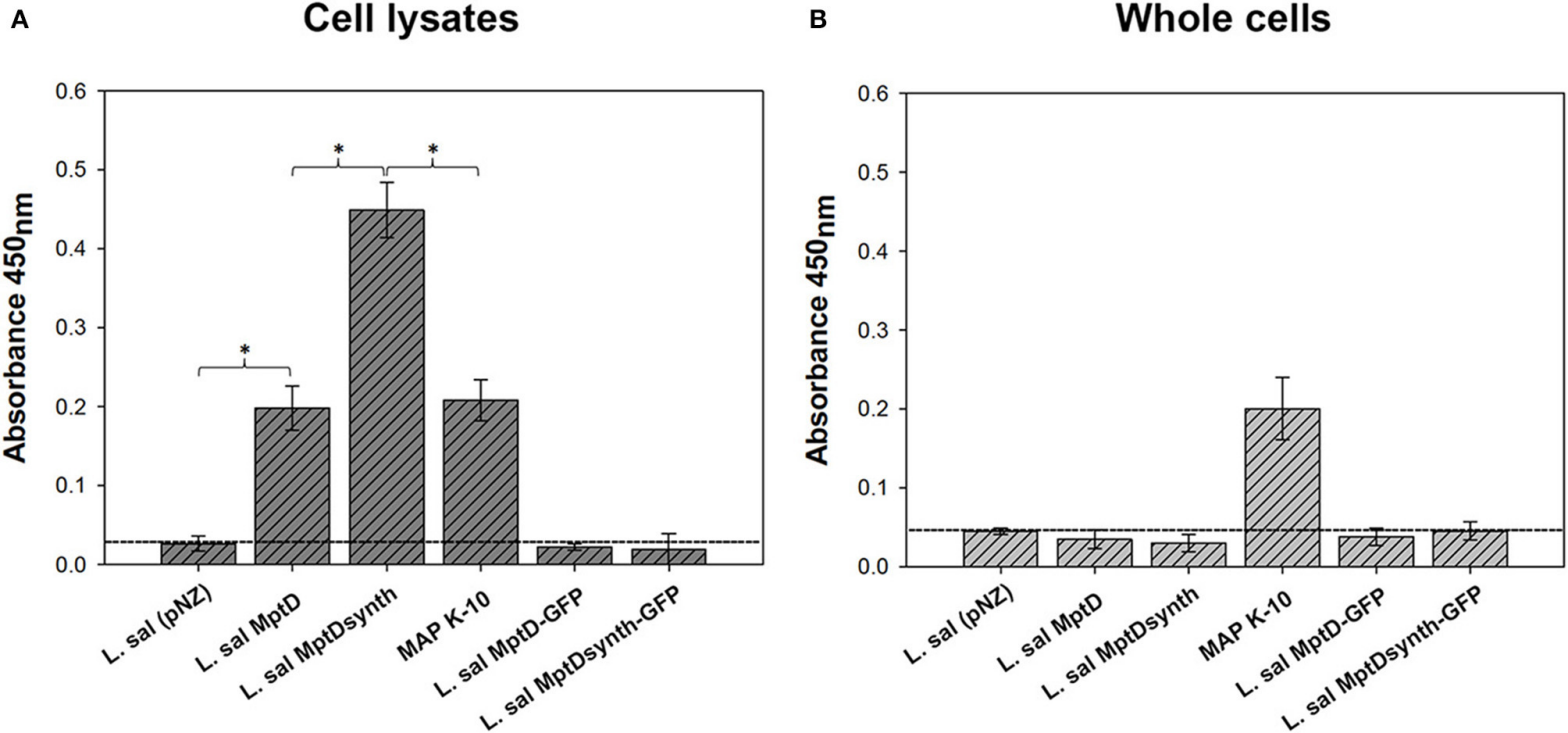
**Comparative fMptD-mediated ELISA analysis of recombinant *L. salivarius* cell lysate and whole-cell against MAP K-10 (A,B)**. Standard deviation of triplicate results is indicated by error bars. Statistically significant difference was observed at ^*^P < 0.05. Horizontal dashed lines indicate the negative control threshold for each assay.

To investigate whether the *L. salivarius* expressed MptD protein was analogous to the native MAP protein, we utilized a modified version of the fMptD bacteriophage mediated ELISA protocol outlined by Rosu et al. (2009), to probe both whole-cell and cell-lysates of recombinant *L. salivarius* transformed with both MptD-GFP gene constructs. Two constructs lacking the GFP fusion were also included to control for potential interference from the tag (Figures 2A,B).

Investigation of the crude cell lysates (Figure 2A) from recombinant *L. salivarius* revealed MptD phage binding to both native and codon optimized MptD constructs lacking a GFP fusion tag with a 2.2-fold increase in signal observed for the codon optimized variant over native (*P* = 0.0008), and 2-fold when compared to MAP K-10 (*P* = 0.0007). Interestingly, in a manner similar to MMP analysis, the addition of a GFP fusion to each of these constructs appears to have effectively inhibited phage binding, possibly by masking the MptD epitope with GFP or prevention of a conformational structure from forming.

Based on the results from confocal microscopy (Figure 1F), we also examined intact cells (Figure 2B) to determine if the tendency of MptD to localize toward the periphery of *L. salivarius* represented true surface exposure. However, despite abundant concentrations of MptD detected within the cytoplasm, no signal (*P* = <0.05 vs. control) could be detected from unbroken cells expressing either native or re-engineered MptD in the presence or absence of a GFP tag. MptD was detected at similar levels in MAP regardless of the cell preparation (Figures 2A,B).

### Re-engineered MMP displays two distinct epitopes

The aggregation observed during confocal microscopy of MMPsynth-GFP prompted further investigation of MMP folding within the *L. salivarius* host. To examine the possibility that the C-terminus GFP tag was itself associating with the MMP peptide, potentially leading to the fluorescent aggregates observed (Figure 1D): two additional constructs, *L. salivarius* with native and codon optimized MMP genes lacking a GFP fusion (pNZ:MAP2121c and pNZ:MAP2121synth), were included in the assays.

Antigenic determinants or epitopes which are recognized on a target protein by an antibody can exist in multiple forms ranging from linear, present on both native or misfolded peptides, to discontinuous or conformationally complex epitopes which are displayed through the native folding of a protein (Brown et al., 2011). In this study, ELISA analysis was performed using two monoclonal antibodies specific to the MMP protein (8G2 and 13E1), which detected two distinct epitopes (Bannantine et al., 2007).

#### ELISA analysis with mAb 8G2 (linear epitope)

**Figure 3:**
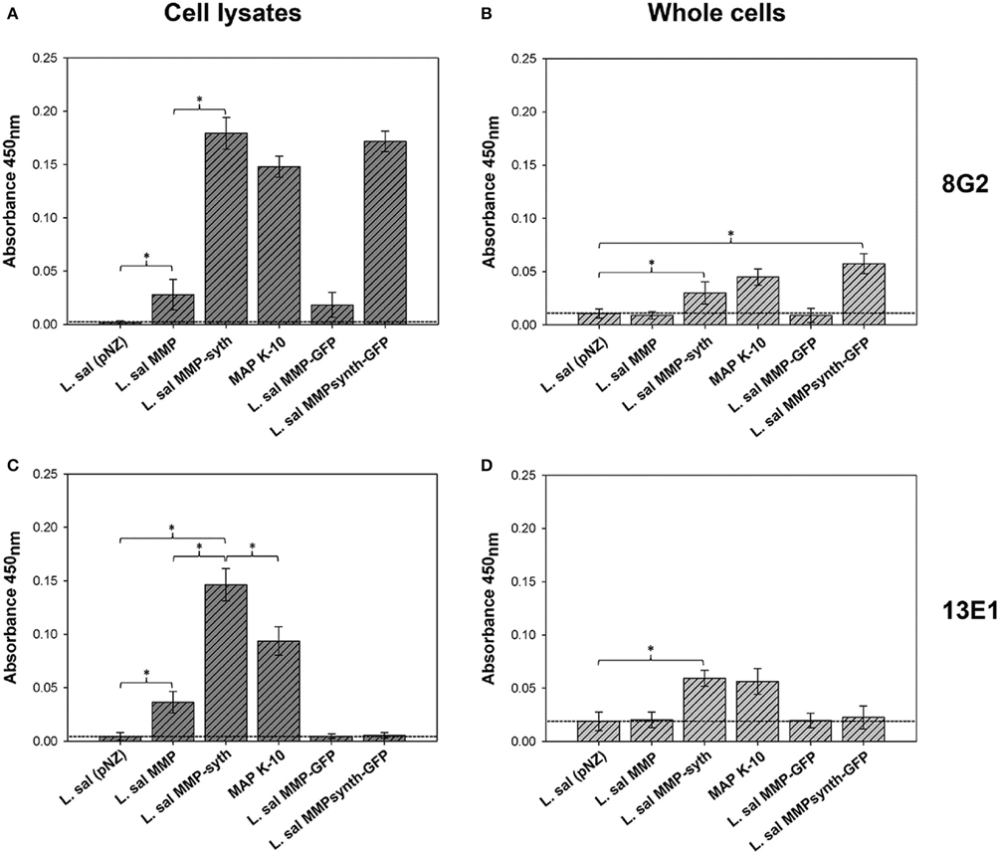
**Comparative ELISA analysis of recombinant *L. salivarius* cell-lysate and whole-cell against MAP K-10, using two monoclonal antibodies directed against MMP**. The 8G2 antibody detects MMP **(A,B)**. The mAb13E1 antibody detects a discontinuous epitope on MMP (**C,D)**. The average value of *L. sal* MMP-GFP and *L. sal* MMP are below the negative control threshold. Standard deviation of triplicate results is indicated by error bars. Statistically significant difference was observed at ^*^P < 0.05. Horizontal dashed lines indicate the negative control threshold for each assay.

The 8G2 antibody is reported to associate with a linear epitope present within a 77-amino acid sequence near the N-terminus of MMP (Bannantine et al., 2007). ELISA analysis with 8G2 on crude cell lysates of recombinant *L. salivarius*-MMP constructs revealed that the linear epitope could be detected in all MMP constructs (Figure 3A), with a significant 6.4-fold signal increase noted for the engineered MMPsynth compared to native MMP.

While microscopy data indicate that heterologously expressed MMP largely accumulates in the cytosol (Figure 1D), intriguingly, the engineered MMP was also detected during whole-cell ELISA using the 8G2 antibody (Figure 3B). Although these levels were low, the signal detected was approximate to that of the MAP K-10 control strain (Figure 3B). Notably, as MMP expression is enhanced in *L. salivarius*, more protein is localized on the surface (Figure 3B).

#### ELISA analysis with mAb 13E1

Analysis of *L. salivarius* cellular lysates with 13E1 indicated that the epitope recognized by this mAb is blocked when MMP is fused to GFP fusions (Figure 3C). However, this epitope appears to be restored by removal of the GFP tag, with 4-fold higher levels of MMP detected from codon optimized MMPsynth when compared to the native gene (*P* = 0.0009), and 1.5-fold when compared to the MAP K-10 control strain (*P* = 0.003).

However, most intriguingly is that MMP protein could also be detected from whole cell *L. salivarius* MMPsynth using the 13E1 antibody, which was comparable to that in MAP K-10, albeit both were present at low levels (Figure 3D). While it has previously been indicated that MMP contains a 30 amino acid hydrophobic domain near the C-terminal end suggestive of a membrane protein, the protein lacks a discernible N-terminal signal sequence (Bannantine et al., 2003). Moreover, it has already been experimentally demonstrated that MMP is both surface exposed and actively shed from live MAP bacilli (Yakes et al., 2008), suggesting that MMP is translocated in a non-classical manner similar to other mycobacterial proteins (Pallen, 2002; Bendtsen et al., 2005).

## Discussion

There have been several efforts at heterologous expression of MAP proteins. *E. coli* expression of an impressive collection of over 650 MAP proteins has been constructed (Bannantine et al., 2010) and these proteins have been incorporated into a protein array to monitor antibody response in different disease stages (Bannantine et al., 2008). Efforts have also been devoted to *in vitro* transcription-translation (Li et al., 2007), but those efforts did not yield much protein to work with. Interestingly MMP was a test protein in that study as well. More recently, MAP proteins have been expressed in a *Salmonella* vaccine delivery strategy (Faisal et al., 2013). Our group is the first to use the probiotic genus *Lactobacillus* as a vaccine vector for MAP. We have overcome the limitations of low expression yields through re-engineering GC-rich coding sequences. No efforts have been made to express MAP proteins in a faster growing species of *Mycobacterium* such as *M. smegmatis.*

We show that MptD, which contains six transmembrane domains, was targeted to the cell periphery, likely the cytoplasmic membrane, while MMP formed aggregates in the cytoplasm of *L. salivarius*. In general, proteins which are located within the cytoplasmic membrane must be targeted to a translocation site prior to their insertion and/or translocation (Fekkes and Driessen, 1999). However, in the case of MptD, such integral membrane proteins generally do not contain a signal sequence. Instead, their hydrophobic TMSs function as an internal signal for targeting and insertion which needs to be recognized early in the translocation pathway to prevent their aggregation in the cytoplasm (Fekkes and Driessen, 1999).

It is known that addition of any fusion tag to the N- or C- termini of a peptide can modify specific structural characteristics either by sterically interfering with protein interactions or disrupting conformational folding (Snapp, 2009). The lack of 13E1 antibody recognition formed by MMP-GFP fusion led us to propose that the fluorescent foci within *L. salivarius* MMPsynth-GFP were related to a masking effect of the MMP epitope with GFP and not representative of translational errors brought about through codon optimization. The GFP tag does not appear to impede localization of MMP at the cell surface, suggesting that this effect is steric at the C-terminus only and does not provide clues on secondary folding that might be required for localization. In originally isolating the 13E1 antibody, Bannantine and coworkers immunized mice with an N-terminal maltose-binding protein fusion with MMP (Bannantine et al., 2007). This suggests that the addition of a large (~40 kDa) protein tag at the N- terminus does not structurally impede the epitope of MMP. However, in our study GFP was at the C-terminus and while it is possible that the GFP tag might mask the 13E1 epitope irrespective of its fused location, it is also possible that the C-terminus is more heavily involved than the N-terminal region in producing the epitope. In support of this, Li et al. (2007) expressed the MMP protein with a six-histidine tag at the C-terminus, noting that the recombinant antigen could not be detected by MAP antibodies in pooled positive serum samples from cattle shedding MAP bacilli (Li et al., 2007).

Detailed studies have demonstrated that MMP is a surface-located virulence factor involved in mediating the invasion of bovine epithelial cells and is transcriptionally upregulated in oxygen limiting and solute stress conditions similar to those encountered within the intestine (Bannantine et al., 2003; Wu et al., 2007). Based upon *in silico* analysis MMP is structurally dissimilar to MptD and analysis lacks polytopic transmembrane domains or highly hydrophobic stretches. The aggregates observed were initially considered to be indicative of translational disorder through synonymous codon modification, since MMP expression in native MAP appears throughout the bacilli by electron microscopy (Bannantine et al., 2003). Alternatively, aggregation could be a consequence of *L. salivarius* reacting to over expression by inducing vesicle formation to compensate for inefficient processing or secretion of a non-host protein. Unfortunately, the negligible fluorescence for native MMP-GFP prevented direct comparison to determine if these foci also occurred in the absence of synonymous mutations necessitating use of the monoclonal antibody based ELISA.

The detection of a non-classically exported protein, such as MMP, from the extracellular surface of *L. salivarius* could indeed be attributed to cell lysis during experimental handling. However, this would presumably have also occurred for those *L. salivarius* cells expressing recombinant MptD, yet this was not observed (Figure 2). Nevertheless, we acknowledge the behavior of the MMP recombinant strains may be different than the MptD recombinants and despite the lack of fMptD phage binding in whole cells, we cannot rule out cell lysis in the whole cell experiments with the MMP strains. As such, we have yet to determine the mechanism by which MMP was effectively presented in such a manner from our heterologous host strain.

Generally, there are three strategies available for the subcellular distribution of recombinant antigens from *Lactobacillus* based vaccine hosts and while cell surface display and secretion are favored, these can result in degradation of recombinant peptides due to exposure to proteolytic enzymes associated with gastric and pancreatic fluids (Kajikawa et al., 2011). Cytoplasmic expression on the other hand, subverts this and protects heterologously expressed peptides from degradation by encapsulation within the cytosol, as well as facilitating the accumulation of high concentrations of the antigenic component intracellularly (de Ruyter et al., 1996). As such, our *L. salivarius* MMPsynth host may provide an interesting combination of both high concentrations of cytoplasmically accumulated MMP, with the added advantage of superficial surface exposure.

Additionally, these results further demonstrate that heterologously expressed codon optimized MptD retains fMptD epitope presentation in *L. salivarius*. However, ELISA data also suggests that MptD, or at least the epitope recognized by the fMptD phage, is not exposed on the surface of *L. salivarius.* It has already been shown that MptD expression on the cell surface of a recombinant host can be achieved using fMptD for both *M. smegmatis* and *M. bovis* BCG (Stratmann et al., 2004; Heinzmann et al., 2008); which could be due to the presence of mycobacterium specific chaperones facilitating appropriate presentation of the MptD protein (Goldstone et al., 2008). However, *mptD* is naturally present on a six gene operon (*mptA-F*) transcribed as a single polycistronic mRNA molecule [60] and in both the aforementioned studies, the recombinant hosts were not transformed with the *mptD* gene in isolation. The *M. smegmatis* harbored a vector including *mptC-F* genes of the mpt operon (Stratmann et al., 2004) while the *M. bovis* BCG host contained the entire operon integrated into the chromosome (Heinzmann et al., 2008). Consequently, it is possible that for effective MptD cell surface display, some ancillary Mpt proteins may also be required. The MptE protein (with five predicted TMSs) encoded by MAP3732c, located immediately downstream and overlapping the MAP3733c gene on the *mpt* operon, is a credible candidate in this respect owing to its association with the C-terminus of the MptD protein.

In future studies it may be interesting to ascertain if the sequential addition of this and other auxiliary *mpt* genes to *L. salivarius* enables native surface display of the MptD protein. Nevertheless, the lack of cell surface display may not be entirely disadvantageous in the context of a MAP antigen delivery host. The association of the MptD protein with the *L. salivarius* membrane, while incomplete, likely sequestered hydrophobic domains thus preventing undesirable aggregation and allowing higher levels of intracellular MptD to accumulate. Moreover it is clear from the analysis of cellular lysate that abundant MptD epitope could be detected within the cytoplasm, indicating that the synthetic gene and the *L. salivarius* host can effectively express and present MptD antigens.

## Conclusion

In conclusion, we have demonstrated that the synonymous mutation of 279 rare or unfavorable codons within the MMP coding region facilitates improved protein synthesis within *L. salivarius*. Furthermore, both synthetic MMP and MptD proteins retain their epitopes or structural characteristics allowing them to effectively mimic the MAP expressed protein. Importantly, we also noted that while codon optimization enhances heterologous overexpression, the addition of a C-terminus GFP tag to both proteins may obstruct some conformational structure from forming.

Nevertheless, in the absence of a GFP tag and any extrinsic signaling peptide, both proteins displayed slight, yet noteworthy, tendencies to associate in the intended location within *L. salivarius*; MMP being detected on the cell surface, while the multi-TMSs containing protein MptD associated with the cytoplasmic membrane boundary. This work underscores the potential of *Lactobacillus salivarius* to be used within a subunit vaccine development against MAP, as additional antigens are optimized for *L. salivarius* expression, the next step will require *in-vivo* testing to demonstrate true efficacy.

## Funding information

The author's acknowledge the financial assistance of the Irish Government through funding under the Food Institutional Research Measure (FIRM) grant 08RDCIT617 as well as the USDA-Agricultural Research Service.

### Conflict of interest statement

The Associate Editor Dr. Adel Talaat declares that despite having collaborated with the author Dr. John Bananntine, the review process was handled objectively and no conflict of interest exists. The authors declare that the research was conducted in the absence of any commercial or financial relationships that could be construed as a potential conflict of interest.
