# Supplementary material for: Enhanced expression of codon optimized Mycobacterium avium subsp. paratuberculosis antigens in Lactobacillus salivarius
Source: Front Cell Infect Microbiol. 2014 Sep 4;4:120. doi: 10.3389/fcimb.2014.00120 (PMC4154528; doi:10.3389/fcimb.2014.00120)
Supplement: Supplementary file 1 [file DataSheet1.DOCX]

| **Supplementary Table S1.** Oligonucleotide primers used in this study | | | | |
| --- | --- | --- | --- | --- |
| *Primer name* | *Primer sequence (5' to 3')* | *Length* | *Characteristics* | *Source* |
| MAP2121c-F | AATTCATGACGTCGGCTCAAAATGAGTC | 28 | (BspHI site) | This work |
| MAP2121c-R | AATAAGCTTTCACTTGTACTCATGGAACTG | 30 | (HindIII site) | This work |
| MAP2121synth-F | AATTCATGACATCAGCACAAAATGAAAGTCAAGC | 34 | (BspHI site) | This work |
| MAP2121synth-R | ATTAAGCTTTCATTTATATTCGTGAAATTGATC | 33 | (HindIII site) | This work |
| MAP3733c-F | AATTCATGACGGCCACTAGCTCGACGAC | 28 | (BspHI site) | This work |
| MAP3733c-R | TTAAAGCTTTCAAGCTAGGCCGGCCCTCTGAA | 32 | (HindIII site) | This work |
| MAP3733synth-F | AATTCATGACAGCAACAAGTAGTACAACACAAAG | 34 | (BspHI site) | This work |
| MAP3733synth-R | ATTAAGCTTTCAAGCTAAACCTGCACGTTG | 30 | (HindIII site) | This work |
| MAP21-R | TCCAGCTGCCTTGTACTCATGGAACTGATCCACC | 34 | Contains 3' Half Linker | This work |
| MAP21-R-ext | TTTTTGAGAACCTCCAGCTGCCTTGTACTCATGG | 34 | Contains 3' Full Linker | This work |
| MAP21synth-R | TCCAGCTGCTTTATATTCGTGAAATTGATCTACAG | 35 | Contains 3' Half Linker | This work |
| MAP21synth-R-ext | TTTTTGAGAACCTCCAGCTGCTTTATATTCGTGAA | 35 | Contains 3' Full Linker | This work |
| GFP-F | TTCTCAAAAAATGTCAAAAGGAGAAGAATTATT | 33 | Contains 5' Half Linker | ([Johnston et al., 2013](#_ENREF_29)) |
| GFP-F-ext | GCAGCTGGAGGTTCTCAAAAAATGTCAAAAGG | 32 | Contains 5' Full Linker | ([Johnston et al., 2013](#_ENREF_29)) |
| GFP-R | TATAAGCTTTTATTTATATAATTCATCCATTCC | 33 | (HindIII site) | ([Johnston et al., 2013](#_ENREF_29)) |
| MCS-F | AACAGTCTTAATTCTATCTTGAGAAAG | 27 | Amplifies across pNZ8048 MCS | ([Johnston et al., 2013](#_ENREF_29)) |
| MCS-R | AAGGTGATTTTTATTTATAAATTAC | 25 |  | ([Johnston et al., 2013](#_ENREF_29)) |
