# Supplementary figures and images for: Enhanced expression of codon optimized Mycobacterium avium subsp. paratuberculosis antigens in Lactobacillus salivarius
Source: Front Cell Infect Microbiol. 2014 Sep 4;4:120. doi: 10.3389/fcimb.2014.00120 (PMC4154528; doi:10.3389/fcimb.2014.00120)

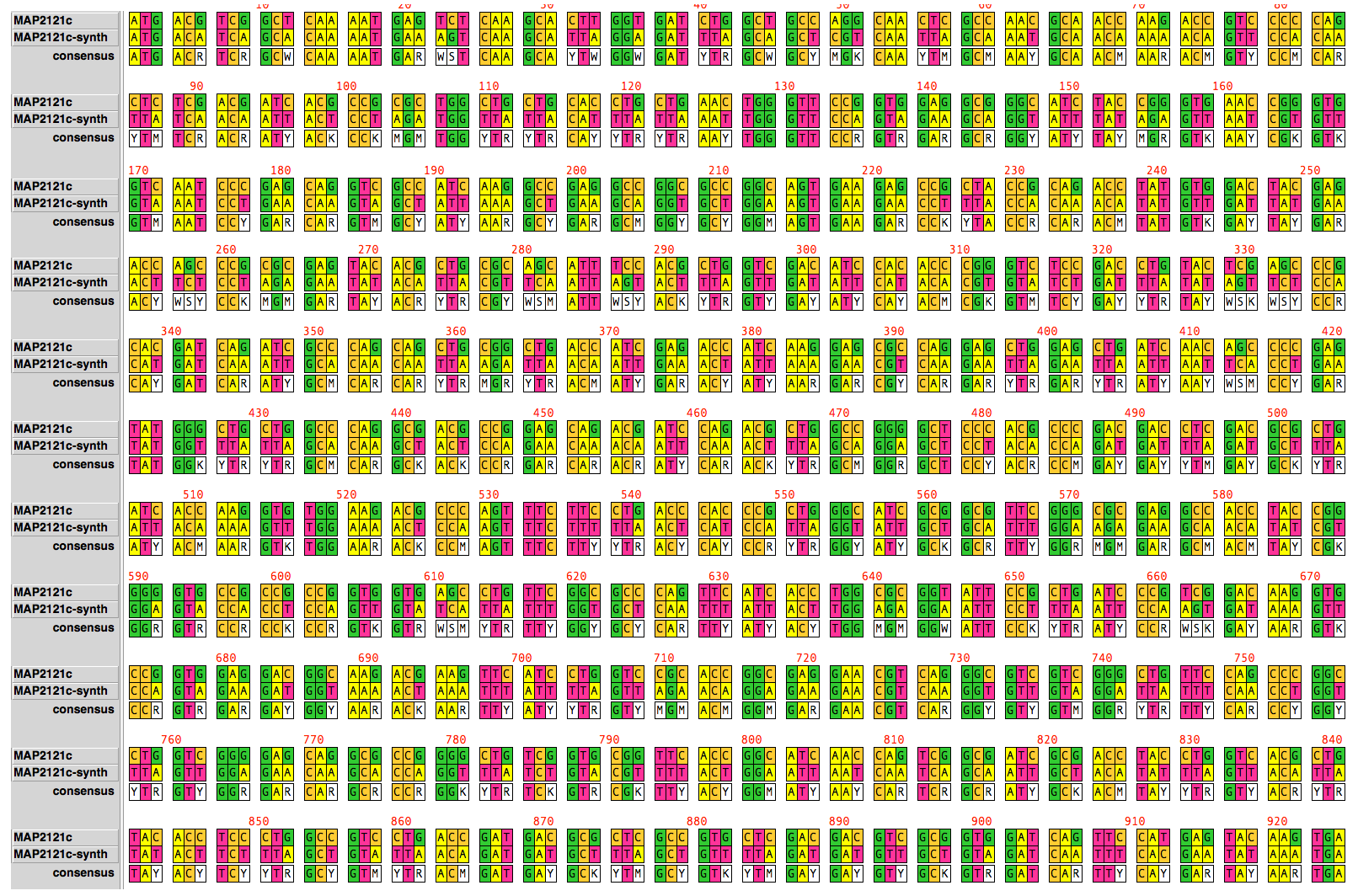

Supplement: Supplementary file 2 [file Image1.TIF]
